# *Big data* e intervalos de referencia: motivación, prácticas actuales, prerrequisitos de armonización y estandarización y futuras perspectivas en el cálculo de intervalos de referencia mediante métodos indirectos

**DOI:** 10.1515/almed-2020-0084

**Published:** 2020-10-30

**Authors:** Luisa Martínez-Sánchez, Fernando Marques-García, Yesim Ozarda, Albert Blanco, Nannette Brouwer, Francesca Canalias, Christa Cobbaert, Marc Thelen, Wendy den Elzen

**Affiliations:** Servicio de Bioquímica Clínica, Hospital Universitario Vall d’Hebron, Barcelona, España; Departamento de Química Clínica y Medicina de Laboratorio, Leiden University Medical Centre, Leiden, Holanda; Departament de Bioquímica i Biologia Molecular, Universitat Autònoma de Barcelona, Bellaterra, España; Departamento de Bioquímica Clínica, Hospital Universitario de Salamanca, Salamanca, España; Departamento de Bioquímica Médica, Facultad de Medicina de la Universidad de Uluag, Bursa, Turquía; Diagnost-IQ, Expert Centre for Clinical Chemistry, Purmerend, Holanda; Laboratori de Referència d’Enzimologia Clínica, Departament de Bioquímica i Biologia Molecular, Universitat Autònoma de Barcelona, Bellaterra, España; Laboratorio de Química Clínica y Hematología, Amphia, Breda, Holanda; Kwaliteitsbewaking Medische Laboratoriumdiagnostiek, Nijmegen, Holanda

**Keywords:** *big data*, intervalos de referencia, métodos indirectos

## Abstract

Los intervalos de referencia son habitualmente empleados como herramienta de apoyo a las decisiones clínicas. En esta revisión se resumen los aspectos relacionados con el *big data* y los intervalos de referencia, las prácticas actuales, incluyendo los métodos estadísticos, los requisitos de calidad de los datos, incluyendo la armonización y la normalización, y las perspectivas de futuro para la determinación indirecta de intervalos de referencia mediante datos de laboratorio de rutina.

## Introducción

Los intervalos de referencia se suelen emplear como herramienta de apoyo a las decisiones clínicas [1]. Una de las principales funciones de los especialistas en laboratorio clínico y medicina de laboratorio es ayudar a los facultativos en la interpretación de los resultados analíticos. Habitualmente los intervalos de referencia hacen referencia al 95% de los resultados de la población de referencia, se suele definir como la media ± 2 veces la desviación estándar (SD) o como los percentiles 0.025 y 0.975 de la población sana [2], [3]. Como resultado, por definición, el 5% de los resultados de individuos sanos quedarán fuera del intervalo de referencia.

Por diferentes razones, existen variaciones intraindividuales e interindividuales de los resultados analíticos por diferentes razones como procesos fisiológicos, diferencias genéticas, factores ambientales o patologías [4]. A la hora de calcular, interpretar y comunicar intervalos de referencia, es importante tener en cuenta dichos factores. La calidad de los intervalos de referencia es tan relevante a la hora de interpretar los resultados como los propios resultados [5]. De este modo, es necesario que los especialistas en laboratorio clínico estén muy familiarizados con los intervalos de referencia, sepan cómo obtener intervalos fiables, y conozcan la evolución de estas estrategias. La cuestión principal es, como expertos, cómo podemos mejorar esta herramienta para permitir una interpretación más sencilla y exacta los resultados analíticos.

Las primeras recomendaciones oficiales sobre la teoría y generación de valores de referencia fueron publicadas por la Federación Internacional de Química Clínica y Medicina de Laboratorio (IFCC) en 1978 [6]. Algunos años antes, en 1969, se formó un panel de expertos con el fin de establecer la primera recomendación en la que, por primera vez, se definió y empleó el término “intervalo de referencia”, en contraposición al ambiguo concepto de “normal” [7]. Tras 1978, basándose en las recomendaciones internacionales, otras sociedades científicas publicaron sus propias recomendaciones [francesa (Société Française de Biologie Clinique, SFBC) [8], española (Sociedad Española de Medicina de Laboratorio, SEQC^ML^) [9], escandinava (Scandinavian Society for Clinical Chemistry) [10], etc]. En 2005, bajo el auspicio de la IFCC, se creó el Comité de Intervalos de Referencia y Valores de Decisión Clínica (C-RIDL). Así mismo, en 2010, el Instituto de Estándares de Laboratorio Clínico (CLSI) publicó una *Guía definitiva para la definición, establecimiento y verificación de intervalos de referencia* basados en las recomendaciones originales [2], [3]. Este documento ha sido ampliamente utilizado para obtener intervalos de referencia mediante lo que conocemos como "método directo".

Según la recomendación del CLSI [2], [11], los intervalos de referencia se deben calcular seleccionando un mínimo de 120 sujetos sanos para poder calcular intervalos de confianza al 90% [12]. Estos se deben seleccionar una vez conocidas las características de la población sana de referencia. Tras seleccionarlos y verificar que su salud es buena mediante cuestionarios y pruebas médicas y/o físicas, se realiza la extracción de sangre, normalmente en el laboratorio. A continuación, se analizan las muestras y, cuando se dispone de todos los resultados, se calculan los intervalos de referencia mediante un análisis estadístico. Las ventajas de este método son las siguientes: (1) el grupo de referencia está adecuadamente caracterizado y controlado; (2) se pueden emplear métodos estadísticos sencillos para calcular los intervalos de referencia directos (por ejemplo, un método no paramétrico); (3) la definición de valores de referencia y el protocolo están estandarizados. Como posibles desventajas: (1) se puede producir un sesgo de selección, debido a la complejidad que entraña seleccionar 120 sujetos sanos de manera aleatoria, contactarlos y reclutarlos (sesgo muestral). Esto significa que, a la hora de contactar con 120 sujetos de forma aleatoria, inevitablemente se incurrirá en un sesgo al decidir cómo seleccionarlos (por ejemplo seleccionando más individuos en algunos barrios en los que se espera una mejor respuesta), la forma de contactar con ellos (por ejemplo por teléfono, mensaje de texto, redes sociales …) y sobre el tipo de personas que se incluirá en un estudio (por ejemplo el horario en el que deben acudir al hospital puede favorecer a algunos grupos frente a otros); (2) las condiciones preanalíticas pueden no reflejar las de la práctica habitual, ya que las muestras extraídas en atención primaria son sometidas a transporte; (3) no es viable determinar intervalos de referencia por grupos de edad/sexo para aquellas pruebas que varían en función de éstos, como la creatinina sérica, que aumenta rápidamente con la edad y difiere entre hombres y mujeres; (4) términos como “población de referencia” y “salud” son subjetivos, y resulta difícil definir las características de un sujeto sano; (5) es un proceso complejo que conlleva más tiempo, recursos y costes; (7) no es viable para algunas pruebas en determinadas matrices, como el líquido cefalorraquídeo, pleural, peritoneal o sinovial, que son difíciles de obtener en individuos sanos, o en algunas poblaciones como la pediátrica.

La progresiva automatización de los laboratorios clínicos ha proporcionado una mayor capacidad de procesamiento, permitiendo así a laboratorios pequeños fusionarse en un único laboratorio con sistemas altamente automatizados. Esto ha resultado en la centralización de los resultados analíticos de extensas áreas geográficas en un solo sistema de información de laboratorio, con una misma estructura de datos fácilmente exportables. Los métodos indirectos se postulan como una alternativa adecuada al cálculo de intervalos de referencia, ya que resuelve muchos de los inconvenientes de los métodos directos [13]. No obstante, los métodos indirectos presentan algunas limitaciones que debemos considerar: (1) el posible efecto de subgrupos no sanos de población en los intervalos de referencia; (2) aún no existe un método para comprobar si los intervalos de referencia obtenidos son correctos y válidos; (3) se pueden producir errores derivados de variación preanalítica o analítica (por ejemplo, cambios metodológicos, cambio de lote de calibradores o reactivos, problemas con el control de calidad); (4) aunque se han propuesto varios métodos estadísticos, aún no existen recomendaciones oficiales sobre qué método utilizar y cuándo utilizarlos.

Otro aspecto a tener en cuenta es la transferencia de los intervalos de referencia. Según el CLSI EP28-A3c [2], para aplicar unos intervalos de referencia a una población diferente a la población para la cual fueron calculados, es necesario verificar que dichos intervalos son extrapolables a otras poblaciones. Esto significa que el laboratorio deberá determinar si la población con la que se calcularon los intervalos es suficientemente representativa de la población en la que estos se quieren utilizar. Estas consideraciones pueden ser relevantes para las magnitudes en las que existen diferencias significativas entre poblaciones, aunque no deben ser nunca un falso argumento para elegir intervalos locales calculados con poca rigurosidad, en lugar de intervalos obtenidos con un método indirecto sólido en una población con diferencias mínimas o potenciales con respecto a la población original. La aplicación del método indirecto podría resolver este aspecto, permitiendo a cada laboratorio calcular sus propios intervalos de referencia basándose en su propia población.

En esta revisión se resumen aspectos relacionados con el *big data* y los intervalos de referencia, describiendo las prácticas actuales, requisitos y perspectivas de la determinación indirecta de los intervalos de referencia mediante datos del laboratorio de rutina.

## Cómo utilizar el *big data* para calcular intervalos de referencia

Los métodos indirectos han ganado popularidad en los últimos años, ya que los datos analíticos masivos son más accesibles actualmente. La definición de análisis de *big data* se basa en el volumen y tamaño del conjunto de datos [14]. Según los MeSH (*Medical Subject Headings*), desde 2019 se define “*big data*” como “*cantidades extremadamente grandes de datos que requieren un análisis automatizado rápido y a menudo complejo para obtener patrones, tendencias y asociaciones, relacionados con distintos aspectos de entidades humanas y no humanas*”. En especialidades médicas, los artículos sobre *big data* suelen emplear una enorme cantidad de individuos y un gran número de variables [14]. Las principales características del *big data* se resumen en las tres uves: volumen (tamaño), variedad (diversidad) y velocidad (frecuencia de actualización) [15]. Algunos autores incluyen una cuarta y quinta uve: veracidad [16] y valorización [17]. Por lo tanto, el conjunto de datos analíticos generados por los laboratorios clínicos podría considerarse *big data* [18]. Además, el uso de la ciencia de datos, definida por MeSH como “*un campo interdisciplinar que implica procesos, teorías, conceptos, herramientas y tecnologías y permite revisar, analizar y extraer un conocimiento y una información valiosa, a partir de datos estructurados y no estructurados*” está ganando terreno en el campo clínico y se espera que lo siga haciendo en los próximos años [19]. Teniendo en cuenta la cantidad de datos sobre sujetos sanos generados en un laboratorio clínico cada día, junto con el desarrollo de nuevas herramientas de ciencia de datos para distinguir a los individuos sanos de los que no lo son, el potencial de estas tecnologías para conseguir unos intervalos de referencia personalizados queda evidenciado. La extrapolación de datos sobre una pequeña muestra de individuos mediante el método directo se transforma en el uso de datos de población real para definir las características de toda la población mediante el método indirecto [20].

No obstante, aún quedan por resolver algunos inconvenientes relacionados con el uso de *big data*:

– La armonización y normalización de las historias clínicas electrónicas: A pesar de esfuerzos internacionales [21], aún quedan por resolver la falta de normalización no solo entre países, sino en los propios países y entre comunidades autónomas o regiones. La armonización de las historias clínicas en un formato común supondría una importante mejora, no solo para la práctica clínica, sino también para estudios retrospectivos y en la calidad de los datos.
– Protección de datos: Algunos conjuntos de datos contienen información sensible [19]. De este modo, aunque la anonimización es esencial, podría suponer un reto, ya que a veces se puede identificar a los individuos por su fecha de nacimiento, sexo, código postal u otras variables [22].

## Breve descripción de los métodos estadísticos: diferencias y similitudes

Mientras que en los métodos directos para establecer valores de referencia la clave es la definición adecuada de la población “normal”, en los métodos indirectos el manejo estadístico de los datos es la parte del proceso más importante a la hora de obtener la mejor información posible a partir del conjunto de datos del que se dispone. En los métodos directos, una vez definida la población “normal” *a priori*, el manejo estadístico de los datos está orientado a decidir qué prueba estadística es la más adecuada. Para ello, se analizan los posibles valores atípicos u *outliers* (por ejemplo, mediante el método de Tukey) y la normalidad de la distribución para seleccionar el método paramétrico (media ±2 desviaciones estándar) o el no paramétrico (percentiles). Existen varios métodos para comprobar la normalidad de la distribución. Debido al pequeño tamaño muestral de los métodos directos (120 sujetos), la opción preferible es la prueba de Shapiro-Wilk, ya que tiene más poder estadístico que la prueba de Kolmogorov-Smirnov [23].

En los métodos indirectos, los datos generados para el diagnóstico y seguimiento clínico de individuos se utilizan (reutilizan) para identificar nueva información (en este caso, para obtener intervalos de referencia poblacionales). Contar con un método estadístico adecuado es esencial en este caso. Para calcular intervalos de referencia mediante el método indirecto, se deben considerar dos aspectos fundamentales: la selección de la población y el manejo estadísticos de los datos.

### Selección de la población

Los aspectos a tener en cuenta son los siguientes [13]:

– Fuente de los datos: Se recomienda emplear datos de pacientes de atención primaria y/o pacientes ambulatorios. Los pacientes hospitalizados padecen patologías, se someten a tratamientos de choque con uso intensivo de soluciones intravenosas, etc. lo cual puede introducir ruido en los datos [13]. Sin embargo, algunos de los métodos nuevos permiten emplear datos de pacientes hospitalizados ya que se pueden detectar y eliminar (automáticamente) los resultados patológicos [24].
– Tamaño de la población: En el método indirecto, esto no supone una limitación, dado el enorme volumen de datos del que se dispone. A pesar de ello, es preferible definir unos mínimos que garanticen la robustez estadística. De acuerdo con el C-RIDL de la IFCC [13], se recomienda emplear al menos 1.000 datos, con al menos 750 datos por categoría (normalmente por sexo y edad) [25].
– Periodo de recopilación de datos: Se recomienda recopilar datos durante al menos un año. De esta forma, se podrá evaluar cualquier posible efecto circadiano o estacional. Además, es importante controlar y monitorizar la estabilidad en el tiempo de los métodos analíticos mediante controles internos y externos (preferiblemente mediante programas externos de garantía de calidad con material conmutable y valores asignados, si los hay) para así reducir la variabilidad causada por cambios en los lotes de reactivos o en los materiales de calibración. Si no se dispone de materiales conmutables externos de calidad, un buen método para comprobar la estabilidad puede ser comparar las medias o medianas diarias, semanales y/o mensuales [26].
– Partición de los datos: Diferentes variables pueden ser consideradas para dividir a la población, como edad, sexo, raza o índice de masa corporal. La edad y el sexo son los elementos de partición más habituales. Es necesario verificar que no existen diferencias entre hombres y mujeres o entre grupos de edad del mismo sexo. En caso de existir diferencias estadística o clínicamente relevantes, se deben establecer intervalos de referencia basados en estos grupos, debido a las implicaciones que estos pueden tener para el manejo clínico de los pacientes. Para determinar si es necesaria la partición de datos, se puede realizar la inspección visual de los diagramas de cajas o aplicar pruebas estadísticas como el análisis de varianza (ANOVA) para la comparación de grupos [27], [28]. Las técnicas de regresión e interpolación cúbica (tal como se describen a continuación) permiten la presentación de intervalos de referencia continuos en lugar de categorías por intervalos de 5 o 10 años de edad.
– Criterios de exclusión (limpieza previa/filtrado de datos): Dependiendo del contexto del laboratorio, puede ser importante eliminar datos de pacientes con alguna patología concreta, algún subgrupo concreto de enfermedades, en tratamiento con algunos fármacos o cuando la extracción de sangre se ha realizado en casa (por ejemplo, cuando los pacientes no pueden acudir al centro de atención primaria debido a su enfermedad). Si se dispone de información sobre el estado fisiopatológico del paciente, ésta será la mejor manera de establecer los criterios de inclusión y exclusión. Sin embargo, cuando no se dispone de dicha información en el sistema de información del laboratorio, se puede emplear otra información presente en la petición analítica (por ejemplo, especialista que solicita la prueba, combinación de pruebas solicitadas mediante protocolos concretos, etc.). Por ejemplo, a la hora de establecer los intervalos de referencia para la creatinina, se podría excluir a los pacientes derivados por el nefrólogo o urólogo, ya que estos pacientes pueden tener alguna patología renal. Como alternativa, en algunos estudios también se excluyen datos de sujetos que tienen múltiples resultados analíticos [29], ya que ello podría indicar la existencia de alguna patología que requiere seguimiento, pudiendo así introducir algún sesgo en los intervalos de referencia calculados.

Antes de describir la aplicación de métodos estadísticos en este contexto, hay que tener en cuenta dos aspectos:

– En lo relativo a los datos de atención primaria (o ambulatorios), un número significativo de estos individuos estarán sanos. Muchas de las determinaciones analíticas se realizan como parte de un chequeo rutinario o para descartar la presencia de una patología (y, en general, pocos resultados serán indicativos de una patología).
– Como normal general, la mayor parte de la población del conjunto total de datos mostrará una distribución normal o casi normal. Este aspecto tendrá que evaluarse según el parámetro estudiado, ya que puede producirse alguna desviación dependiente del parámetro.

### Análisis estadísticos

Este es un factor crucial de los métodos indirectos. En los diferentes estudios descritos en la literatura [24], [29], [30], [31], [32], [33], [34], [35], [36], [37], [38] los métodos estadísticos empleados se pueden agrupar en dos estrategias:

– Grupo A: Basándonos en el conjunto de datos, se aplican técnicas estadísticas para eliminar datos extremos o atípicos (*outliers*), antes de emplear otros métodos estadísticos para calcular los intervalos de referencia.
– Grupo B: Se aplica directamente un método estadístico a todo el conjunto de datos sin eliminar ninguno previamente para calcular los intervalos de referencia.

#### Grupo A

Cuando se emplea una base de datos global de rutina, existen valores analíticos de individuos sanos y enfermos. Los valores patológicos que se encuentran en los extremos de la distribución de los datos influirán en los intervalos de referencia calculados mediante métodos estadísticos estándar. En la literatura se han descrito diferentes estrategias para eliminar los valores atípicos. Zellner et al. compararon recientemente los diferentes métodos para la eliminación de valores atípicos y concluyeron que el test de Tukey es el más apropiado en la determinación de intervalos de referencia [39].

Esta metodología del grupo A se basa en la premisa de que los datos atípicos eliminados corresponden a individuos no sanos, mientras que los datos restantes corresponden mayoritariamente a individuos sanos. Esta situación es muy común en las bases de datos de rutina, dado que normalmente los valores de los individuos sanos y no sanos se suelen solapar [24] aunque dependiendo de la magnitud, esta premisa puede llevar a errores. Así, para los test con un índice bajo de individualidad (división entre variabilidad interindividual, CV_I_ y variabilidad intraindividual, CV_G_), existe un alto grado de solapamiento entre los individuos sanos y no sanos, no pudiendo eliminar adecuadamente a las dos poblaciones mediante los métodos de eliminación de outliers. De este modo, los datos de la población no sana que no han sido eliminados pueden influir en los valores de la población sana, afectando a los intervalos de referencia obtenidos. Los autores del proyecto NUMBER emplearon el método de Tukey para eliminar los valores atípicos haciendo uso de magnitudes bioquímicas relacionadas [34], tratando de excluir los datos de poblaciones potencialmente enfermas. Futuros estudios podrían establecer la influencia real de las poblaciones con patologías en los resultados de intervalos de referencia.

#### Grupo B

Los datos de individuos sanos y no sanos muestran cierto grado de solapamiento, lo cual depende del tipo de test. Basado en esta premisa, se han aplicado métodos estadísticos a las bases de datos de laboratorio, que permiten separar estas dos poblaciones adecuadamente. Para tal fin, existen dos métodos tradicionales basados en estrategias gráficas: el método Hoffmann y el método Bhattacharya [13]. En ambos métodos se trata de identificar a una población normal en el total de la población identificándola como población sana. El objetivo es caracterizar a la mayor parte de la distribución central de todos los datos, representando a la población sana. En estos métodos, la parte central se define mediante puntos de truncamiento. En las bases de datos en las que, además de la población de sujetos sanos, la otra población de individuos (normalmente con patologías) es de un tamaño significativo, la segunda influirá de manera negativa en la determinación de intervalos de referencia cuando se aplica el método Hoffmann. Sin embargo, esta población de pacientes influye menos en el método Bhattacharya [13]. Una limitación importante del método Bhattacharya es la influencia subjetiva del resultado obtenido, ya que es necesario definir el tamaño de los intervalos (*bin size data*), la localización y el número de intervalos empleados en cada conjunto de datos. En estos dos métodos, la representación gráfica de los datos juega un papel fundamental a la hora de estimar los intervalos de referencia, aunque esta no es necesaria con el método Bhattacharya. En un estudio comparativo entre el método indirecto de Bhattacharya y método directo recomendado por la IFCC, publicado en 1990 [40] se mostraron importantes diferencias en los intervalos de referencia obtenidos. El estudio reveló que las diferencias halladas se debían a los métodos estadísticos empleado y no solo a la población de referencia, y que dichas diferencias dependen además de la forma de la distribución.

Arzideh et al. [33] propusieron un método alternativo (*Truncated Maximum Likelihood*) a los tradicionales métodos de Hoffmann y Bhattacharya, en el que se considera que la población sana muestra una distribución normal, mientras que la población no sana muestra otro tipo de distribución. Se estiman las funciones de densidad no paramétricas para analizar la distribución de todos los grupos de la muestra (población sana y no sana combinada) mediante la estimación de densidad suavizada de Kernel. A continuación, se obtienen dos funciones de densidad: una para la población sana y otra para lo población no sana. La desviación con respecto a la distribución normal se detecta mediante un test de bondad de ajuste, identificando así a la población no sana. Finalmente, los puntos de intersección entre la función de densidad (sanos y no sanos) muestran el valor de los intervalos de referencia (Zierk et al. [23]) emplearon esta técnica para obtener intervalos de referencia para cada grupo de edad y los combinaron mediante la técnica de *splines* (*cubic smoothing spline, spline* cúbico suavizado) para generar intervalos de referencia continuos.

Recientemente, Wosniok y Haeckel propusieron un método alternativo: el chi-cuadrado mínimo truncado (*TMC*). Para este método, los parámetros de la hipotética distribución normal se calculan de forma preliminar representando los datos en un gráfico Q-Q. El método *TMC* presupone que los datos de los sujetos sanos sigue una distribución normal y, empleando el chi-cuadrado mínimo, se calcula la media (*μ*), la desviación típica (*σ*), la varianza (*λ*) y la bondad de ajuste de esta distribución para cada intervalo truncado definido por el modelo. Los valores *μ, σ, λ* del intervalo con la mejor bondad de ajuste se emplean para calcular los intervalos de referencia (al 95%). Antes de calcular los intervalos de referencia, la población se estratifica en grupos de edad y, una vez calculados, se combinan empleando la técnica de *splines* descrita anteriormente.

Las técnicas de este grupo permiten separar las poblaciones sanas de las no sanas de forma fiable, aunque sean más difíciles de aplicar e interpretar.

## Requisitos previos

Para mejorar la interpretación de los informes analíticos en todo el mundo, es necesario que la información sea comparable [41], por tanto, se recomienda la armonización de los informes emitidos por los laboratorios clínicos. Para tal fin, es importante tener en cuenta todos los aspectos del proceso, no solo los aspectos preanalíticos y analíticos, sino también la nomenclatura, terminología, unidades, formato, intervalos de referencia y valores de decisión clínica [41], [42].

Las variaciones en los intervalos de referencia entre laboratorios clínicos afecta directamente a los pacientes, llevando a la disparidad en las interpretaciones clínicas de los mismos resultados, o a la repetición innecesaria de las pruebas analíticas [43], [44]. Esta realidad ha adquirido mayor importancia en la actualidad, dada la creciente movilidad de la población (dentro del país) y las múltiples consultas a diferentes médicos en contextos clínicos variados. Los sistemas nacionales o autonómicos de historias clínicas electrónicas de atención primaria reciben resultados de diferentes laboratorios [45]. La armonización de los intervalos de referencia, obtenidos mediante métodos indirectos de *data-mining* facilitará el intercambio de datos estandarizados entre los sistemas de salud, contribuyendo así a reducir la repetición innecesaria de pruebas analíticas por parte de diferentes facultativos en contextos clínicos diferentes.

La estandarización de las unidades en las que se expresan los resultados es un requisito importante para una aplicación segura de dicha armonización. El empleo de un sistema internacional de unidades es un requisito obvio, aunque parece representar un gran obstáculo en países como EE.UU, Alemania y España. Además, el empleo de un sistema internacional de unidades por sí solo no garantiza la armonización de las mismas. La interpretación errónea de resultados y la mala aplicación de las guías analíticas es un riesgo importante derivado del uso de diferentes unidades entre laboratorios [46], especialmente en entornos geográficamente cerrados.

La armonización/estandarización de las pruebas diagnósticas *in vitro* (*IVD,* por sus siglas en inglés) y la validez de los resultados de las pruebas son requisitos imprescindibles antes de poder extraer datos de un sistema de información del laboratorio, para establecer intervalos de referencia. La regulación europea de *IVD* exige la trazabilidad de los controles y calibradores a métodos de referencia de orden superior y materiales de referencia, cuando sea posible [44] y, por encima de estas, la ISO 17511:2020 [47] exige la trazabilidad de los resultados de las pruebas a sistemas de medición de referencia de orden superior. De este modo, es importante: (1) que las pruebas de laboratorio utilizadas se encuentren estandarizadas por la industria de *IVD* y cumplan las especificaciones; (2) que los especialistas de laboratorio conozcan dichas regulaciones e implementen dichas pruebas normalizadas [48]; y (3) que se empleen materiales conmutables específicos para la verificación de la veracidad. En Holanda, en el programa “Calibration 2000”, el desarrollo de materiales conmutables específicos fueron considerados el “santo Grial” [49]. La implementación del programa de evaluación de calidad externa holandés “SKML Combi New Style” de 2005, demostró que el uso de suero conmutable valorado es muy eficaz a la hora de reducir la mediana de los coeficientes de variación entre laboratorios en la determinación de electrolitos, sustratos y enzimas [50]. Un estudio de comparabilidad entre métodos analíticos llevado a cabo en España empleando los mismos materiales conmutables del SKML ha demostrado que aún no existe una armonización entre laboratorios [51], [52]. Ricos et al. ya recomendaron cambiar a los métodos con piridoxal fosfatopara la determinación de alanina aminotransferasa (ALT) y aspartato aminotransferasa (AST), el uso del método enzimático para la medición de creatinina, cambiar a los métodos de piruvato a lactato para determinación de lactato deshidrogenasa (LDH) y emplear calibradores conmutables para los electrolitos [51]. Es importante recordar estas recomendaciones.

De este modo, los laboratorios clínicos debería cambiar a los métodos recomendados por la IFCC y emplear materiales de calibración conmutables y materiales EQA con valor asignado para: (1) reducir la variabilidad intra e interlaboratorio y mejorar la equivalencia entre métodos [49]; (2) permitir el cálculo y la comparación de intervalos de referencia entre laboratorios empleando el método directo o indirecto; (3) permitir la implementación de intervalos de referencia nacionales o incluso internacionales normalizados; y (4) implementar un sistema de monitorización para los intervalos de referencia comunes establecidos.

## Conclusiones

Los métodos indirectos son una herramienta prometedora para determinar intervalos de referencia específicos, asequibles y actualizados en los laboratorios. Dada la existencia de múltiples métodos estadísticos, recomendamos realizar un estudio comparativo internacional de dichos métodos, con el fin de alcanzar un consenso sobre los criterios a aplicar (selección de la población, limpieza previa de los datos, …) a la hora de determinar qué procedimiento y método estadístico se debe emplear para cada prueba. Para tal fin, recomendamos la realización de una revisión sistemática de la literatura para comparar los resultados obtenidos en los diferentes estudios empleando el método directo e indirecto en la misma población, y poder comparar los resultados de los estudios indirectos aplicando métodos similares. El objetivo último es establecer unos protocolos consenso en los que se recomiende qué método utilizar para cada prueba, cómo comparar los intervalos de referencia obtenidos mediante métodos indirectos y cómo investigar la transferencia entre poblaciones.

Un requisito esencial para el éxito de los métodos indirectos para la determinación de intervalos de referencia y para poder comparar los resultados obtenidos en diferentes laboratorios o diferentes países es adoptar una actitud común hacia el uso únicamente de métodos analíticos estandarizados o armonizados. Los especialistas de laboratorio son agentes clave a la hora de facilitar el empleo de estas estrategias de *big data*, ya que son expertos en determinar qué pruebas analíticas estandarizadas o armonizadas (recomendadas por la IFCC) y qué resultados se pueden utilizar para calcular intervalos de referencia de un laboratorio o combinar los resultados analíticos de varios laboratorios para obtener intervalos de referencia regionales o nacionales.

A pesar de que la guía EP28-A3c del CLSI recomienda el método directo para el cálculo de intervalos de referencia, se debe considerar el método indirecto como un método alternativo no solo para la obtención de intervalos de referencia en los laboratorios locales, sino también para la verificación de los intervalos de referencia utilizados y obtenidos por los métodos directo o los intervalos descritos en los *inserts*. Para armonizar los intervalos de referencia globalmente, los intervalos obtenidos mediante el método directo e indirecto se deben evaluar empleando una metodología basada en la evidencia.
